# Characterization of Pressure Distribution in Penetrating Traumatic Brain Injuries

**DOI:** 10.3389/fneur.2015.00051

**Published:** 2015-03-13

**Authors:** Johan Davidsson, Mårten Risling

**Affiliations:** ^1^Applied Mechanics, Chalmers University of Technology, Göteborg, Sweden; ^2^Department of Neuroscience, Karolinska Institute, Stockholm, Sweden

**Keywords:** penetrating traumatic brain injuries, TBI, pressure, experimental

## Abstract

Severe impacts to the head commonly lead to localized brain damage. Such impacts may also give rise to temporary pressure changes that produce secondary injuries in brain volumes distal to the impact site. Monitoring pressure changes in a clinical setting is difficult; detailed studies into the effect of pressure changes in the brain call for the development and use of animal models. The aim of this study is to characterize the pressure distribution in an animal model of penetrating traumatic brain injuries (pTBI). This data may be used to validate mathematical models of the animal model and to facilitate correlation studies between pressure changes and pathology. Pressure changes were measured in rat brains while subjected to pTBI for a variety of different probe velocities and shapes; pointy, blunt, and flat. Experiments on ballistic gel samples were carried out to study the formation of any temporary cavities. In addition, pressure recordings from the gel experiments were compared to values recorded in the animal experiments. The pTBI generated short lasting pressure changes in the brain tissue; the pressure in the contralateral ventricle (CLV) increased to 8 bar followed by a drop to 0.4 bar when applying flat probes. The pressure changes in the periphery of the probe, in the Cisterna Magna, and the spinal canal, were significantly less than those recorded in the CLV or the vicinity of the skull base. High-speed videos of the gel samples revealed the formation of spherically shaped cavities when flat and spherical probes were applied. Pressure changes in the gel were similar to those recorded in the animals, although amplitudes were lower in the gel samples. We concluded cavity expansion rate rather than cavity size correlated with pressure changes in the gel or brain secondary to probe impact. The new data can serve as validation data for finite element models of the trauma model and the animal and to correlate physical measurements with secondary injuries.

## Introduction

Traumatic brain injury (TBI) is defined as the result of external forces applied to the head and transmitted to the brain, which in turn induce brain tissue responses including a combination of short lasting strain and shear stress and pressure changes (1–3). Once injured the brain response varies vastly from case to case due to the size, age, genetics, and gender of the victim, as well as the characteristics of any external force. Often the brain response results in temporary and limited brain malfunction known as concussion, although a primary injury with permanent brain tissue damage at the cellular level is commonly sustained. Sometimes bleeding, inflammation, hypoxia, and edema follow primary brain injuries. These events may lead to a subsequent increase in intracranial pressure and a negative spiral of further secondary injuries (4).

Since the brain is 75% water, it may be hypothesized secondary injuries may be related to induced cavity formation from impact and pressure wave propagation through the brain tissue (5). Such transient events of an impact cannot be monitored in a clinical setting; the development and use of animal models are needed for further detailed studies into the effect of pressure waves and their relation to subsequent injury. Appropriate models can reduce confounding factors and uncontrollable variations seen in real life situations (6). Proper monitoring of physical brain responses is essential and it is vital that the model is validated in order to ensure it represents the particular injury it is designed to mimic. In theory, experimental models can be analyzed with mathematical models, more specifically with finite element (FE) models, and compared to carefully categorized clinical TBI cases (7, 8). For such an analysis to be useful, the animal FE-models should be thoroughly validated using physical measurement data from relevant animal experiments.

The aim of this study was to provide generic information on the relationship between an object rapidly penetrating the brain and the resulting dynamic pressure changes as recorded in several regions of the brain and spine. We have employed a model for pTBI (9) suitable for examining the consequences of a focal impact at a controlled and varied velocity. We have studied the relationship between the velocity and shape of a penetrating object and the resulting pressure changes in gel samples and in an animal brain. The covering skull bone was removed in this simplified animal model and the model can therefore be entirely focused on any brain tissue response. The study also aimed to define this particular model to enable a correlation study between pressure changes and secondary injuries, as well as to allocate data for validation of FE-models of the trauma model.

## Materials and Methods

This study focused on pressure distribution within animal brains during simulated penetration trauma. For additional understanding of the pressure origin, experiments with gel samples were carried out to study the formation of cavities during simulated trauma.

### Test rig

**Figure 1:**
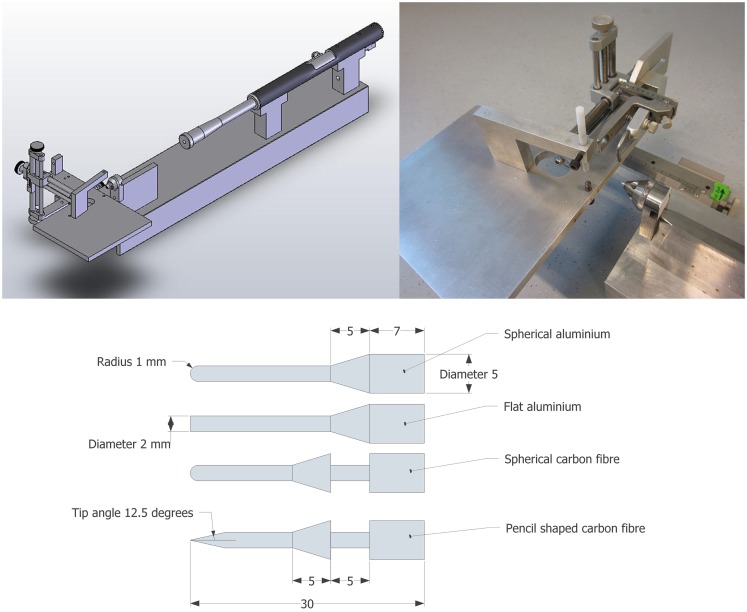
**Schematic of the test device (top left), photo of the stereotactic frame and animal table and the cone designed to guide the probe (top right) and probe designs (bottom); spherical and flat aluminum probes (top two) spherical and Pencil shaped carbon fiber probes (bottom two) (mm)**.

The test rig was presented by Plantman et al. (9). In brief, a lead bullet (Accupell, Crossman, Bloomfield, NY, USA) with a mass of 0.924 g was accelerated by air pressure from a specially designed air driven rifle (Provvapen, CNC-process AB, Hova, Sweden) and made to impact a probe. The probe, guided by a probe holder, penetrated the brain of the animal held in position in a purposely built stereotactic frame (Figure 1). In past experiments, the probe consisted of a metal cylinder with an attached carbon fiber pin with a tip radius of 1 mm. By use of a special brass cuff fitted around the probe, the depth of penetration was limited to approximately 5.5 mm. This probe design, referred to as the carbon fiber probe, was used for five tests in this study (test 288–295). For all other experiments (*n* = 22) presented in this study a more recent probe design was used; the probe was constructed in one single unit in aluminum. The complete weight of the carbon fiber probe, including the brass cuff, was 1.54 g while the weight of the aluminum probe was 0.66 g. The diameter of the penetrating pin of all probes was 2 mm. The tip that penetrated the brain was either flat, pencil shaped, or spherical (Figure 1).

The same test rig was used for the experiments with gel samples, with the exception that some devices were used to rigidly secure the gel samples.

#### Lead bullet and probe velocities

A chronograph (SKAN PRO1 Series 3, SkanAR, Essex, England, UK) was used to capture the velocity of the lead bullet in a separate series of experiments.

The motion of the carbon fiber probe penetrating the brain was recorded by a MotionPro HS-4 high-speed video (Redlake MASD, Inc., San Diego, CA, USA) at 200,000 fps (8 × 512 pixels). The motion of the aluminum probe penetrating the brain was recorded by a FASTCAM SA-X2 type 1000K-M2 camera (Photron USA Inc., San Diego, CA, USA) at 100,000 fps (320 × 128 pixels). A Sigma 105 mm macro lens (reproduction ratio of 1:1.5, Sigma Corporation, Kabushiki Gaisha Shiguma, Japan) was used in both videos. Film analysis was carried out using TEMA Automotive 3.5-012 (Image Systems AB, Linköping, Sweden) to estimate the probe velocity.

### Animal studies

#### Animals and surgery

In a total of 27 Sprague-Dawley rats weighing between 320 and 590 g were anesthetized by a 2.4 mL/kg intra-abdominal injection consisting of a mixture of 1 mL Midazolam (5 mg/mL), 1 mL Hypnorm (VetaPharma Ltd., Sherburn Enterprise Park, Sherburn-in-Elmet, Leeds, UK), and 2 mL of distilled water. All experiments were performed in accordance with the Swedish National Guidelines for Animal Experiments and were approved by the local ethics committee in Stockholm, approval reference N81/13.

A midline incision was made through the skin and periosteum, which exposed a part of the calvaria bone. Then a burr hole with diameter of 2.75 mm was drilled through the calvaria with its center 2 mm lateral and 2 mm posterior of the bregma. This hole was later used for the introduction of the probe into the brain cavity.

**Figure 2:**
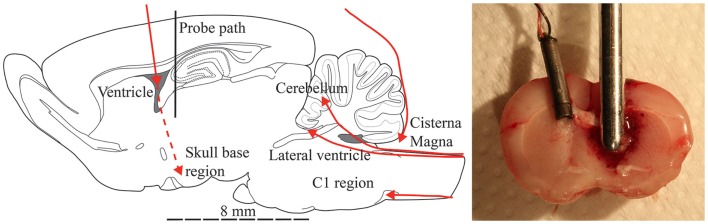
**Schematic sagittal section of a rat brain (lateral 1.90 mm to midline) with red lines depicting the FOPT positions (arrow indicate sensor position) used in the pressure measurements (left). Photos to that illustrate the position of the traditional pressure transducer in experiment 322 and the probe in its final position after trauma (right)**.

Fiber optic pressure transducers (FOPTs) were used in this study. These were guided into the brain through thin polytetrafluoroethylene (PTFE) tubes that were inserted into the brain. Prior to insertion into the brain, the tube was adjusted in length and filled with saline solution. For most animals, a second burr hole, with a diameter of 0.9 mm, was drilled through the calvaria contralateral to the first burr hole (2 mm lateral of the midline suture and 1.5 mm rear of the bregma) and the tube was guided into the contralateral ventricle (CLV, 4 mm below the dura) (Figure 2). In some animals, the same burr hole position was used but the length of tube inserted was only long enough for the tip to make contact with the skull base. The tube was withdrawn 1 mm on contact with the base. In some animals, the second burr hole was drilled through the midline of the occipital bone and a tube was guided to the Cisterna Magna (CM) region.

In a few animals, a 20 mm long midline incision in the T11–L2 region was made. The spine processes were freed from soft tissues and the rear arch of the T12 or L1 and the spinous process of the adjacent vertebra were removed. A small cut was made in the exposed dura and a PTFE tube was guided through the canal until the tube tip reached either the cerebellum (Cb), forth ventricle (4V), or the level of the first cervical vertebra (C1) (Figure 2).

In one animal (Table 1, No 322), an additional burr hole of 1.3 mm in diameter was drilled through the calvaria contralateral to the first burr hole. A piezo-resistive sub-miniature pressure transducer was inserted 3.5 mm into the brain cavity through this hole (Figure 2). The transducer was inserted and secured, and the hole was closed by dental glue (Super-Bond C & B; Sun Medical Co., Shiga, Japan), which was left to cure for 5 min (a fan was used to blow air at a temperature of 37°C at the head).

**Table 1:** **Animal number, weight trauma conditions, and pressure transducers used and their locations**.

No.	Animal weight (g)	Pressure (bar)			Transducer position						Penetration (mm)
		30	50	100	CLV	CM	Skull base	Cb	4V	C1	
**Spherical shaped aluminum probe**
285	355	*			*						5.8
286	467	*			*						5.7
287	321	*			*						5.7
282	393		*		*						5.7
283	425		*		*						5.7
284	469		*		*						5.7
330	344		*		*						6.0
322	554		*		*^a^						5.9
329	359		*			*					5.8
341	357		*			*	*				6.2
342	352		*			*	*				6.4
323	563		*					*			5.9
324	593		*						*		5.9
326	581		*							*	5.9
**Flat shaped aluminum probe**
335	324		*		*	*					6.3
336	338		*		*	*					6.4
337	345		*		*	*					6.2
338	354		*				*			*	6.2
339	342		*				*			*	6.2
340	333		*				*			*	6.3
333	324			*	*						6.1
334	312			*		*					6.1
**Spherical shaped carbon fiber probe**
291	385		*		*						5.0
292	356		*		*						5.2
295	367		*		*						5.1
**Pencil shaped carbon fiber probe**
288	401		*		*						6.9
293	417		*		*						5.8

*Contralateral ventricle (CLV), Cisterna Magna (CM), skull base region, center of the cerebellum (Cb), forth ventricle (4V), and the level of the first cervical vertebra (C1)*.

*^a^Resistive transducer*.

#### Instrumentation and data acquisition

Either one or two FOPTs (extended range 360HP, range 0.11–10.95 bar absolute pressure, Samba Sensors AB, Göteborg, Sweden) with a diameter of 0.36 mm or a resistive transducer with a diameter of 1.27 mm and a length of 6.4 mm (EPIH, range 0–7 bar absolute pressure, Measurement Specialities, Hampton, VA, USA) were used to record pressures.

The lengths of the PTFE tubes were adjusted to ensure the sensor element of the FOPT was 0.5 mm from the end of the tube when the free length of the FOPT fiber was fully inserted into the tube. Hence the tube guided the sensor in position and protected the sensor tip from direct mechanical influence by any tissue.

Fiber optic pressure transducers were connected to a SAMBA 202 control unit (Samba Sensors AB, Göteborg, Sweden) that converted the optical signal to an analog voltage signal. This signal ranged from 0 to 10 V and was updated at 40 kHz. The pressure probes were calibrated to the ambient air pressure immediately before insertion, using the internal calibration system of the control unit. The resistive sensor signal was offset adjusted, amplified, and anti-aliasing filtered (30 kHz Bessel low pass) using a DEWETRON DAQP-Bridge-B signal conditioner (DEWETRON GmbH, Grambach, Austria).

The measurement hardware and the software were updated during the test series and it was deemed necessary to use two measurement configurations.

First, a single measurement signal as provided by the SAMBA 202 unit or the Dewetron unit were digitized using a National Instrument USB-PCMCIA card 6062 set for reference single ended measurements installed in a Hewlett Packard NC2400 computer (Hewlett-Packard Company, Palo Alto, CA, USA). The card was controlled through the software LabView, version 8.5 (National Instruments Corp., Austin, TX, USA). Sample frequency was 100 kHz in experiments 282 through 283; thereafter 200 kHz. An analog voltage sensitive trigger was used.

Second, two measurement signals as provided by the SAMBA 202 unit were digitized by a National Instrument USB-6251 data acquisition unit, set for reference single ended measurements, connected to a DELL laptop (Dell Inc., Round Rock, TX, USA). The unit was controlled through the software LabView version 2013; single ended measurements at a sample frequency of 200 kHz. An analog voltage sensitive trigger was used.

#### Test procedures

After surgery the rats were placed in a stereotactic frame. A syringe was used to flush 0.1 mL saline solution into the PTFE tubes that had previously been inserted into the animals. Thereafter, the FOPT pressure transducers were guided into the tubes (Figure 2). The FOPT were secured in the tube by adhesive tape during the trauma.

The stereotactic frame was positioned so that the impactor probe was located directly above the dura exposed by the burr hole. In addition, the animal heads were aligned so that the probe was perpendicular to the external vault surface. Thereafter, the animals were subjected to one single penetration trauma (probe path indicated in Figure 2). Three pressure levels in the air rifle chamber were used; 30 (*n* = 3), 50 (*n* = 22), and 100 (*n* = 2) bar. Details about the various tests can be found in Table 1.

All animals were euthanized immediately after the trauma. Thereafter, the tube/pressure transducer tracks were inspected visually and documented.

#### Data analysis

One tail and two-sample assuming unequal variances *T*-tests were carried out to identify significant differences (at 0.05 levels) for maximum pressure between treatment groups. Results are reported as mean ± 1 SD unless otherwise indicated.

### Gel sample studies

#### Gel samples

Two types of containers were produced in clear rigid plastic, for cavity formation studies and for pressure measurements (Figure S1 in Supplementary Material). Both types of containers were made to fit the test rig and had a 2.7 mm diameter hole to allow for initial probe to gel contact. 10% nominal concentration gel produced in accordance with Jussila (10). In brief, 30 g of Gelatine SG 720-N (Gelita Sweden AB) was mixed with 120 g of cold tap water. After 2 minutes, 150 g of purified and warm water (60°C) was added to the Gelatine and water mixture and mixed for 4 minutes. Thereafter, the mixture was poured into the containers intended for use in the experiments, tightly wrapped in plastic and immediately transferred to a refrigerator. The containers were stored in the refrigerator for 2–3 h.

#### Instrumentation and data acquisition

For studies into cavity formation, a FASTCAM SA-X2 type 1000K-M2 camera fitted with a Sigma 105 mm macro lens (reproduction ratio of 1:1.5) was used to record the event at 45,000 fps. The image size was 512 × 512 pixels. The camera was controlled via Photron Fastcam Viewer Version 3,3,8,0. A sound trigger (MD1505, Kapture Group Inc., Osage Beach, MO, USA) was used to start the video recordings. Two 250 W COOLT2 lights (Dedolights, Ashley Falls, MA, USA) were used to illuminate the gel sample. Fans ventilated the lights; the air was blown away from the gel samples to avoid heating the samples. For measurements of cavity size, the video images of the event were imported into a CAD program (SketchUp, Google, Inc., Mountain View, CA, USA). Maximum cavity length (in the direction of probe travel) and maximum cavity height (in the direction perpendicular to probe travel) were measured using this software. In addition, the probe front position relative to the center of the cavity was measured. Film analysis was carried out using TEMA Automotive 3.5-012 to estimate the cavity wall velocity.

For pressure measurements in gel samples, three strain gage pressure transducers (XPM5-20BG-ET3 from Measurement Specialities, Hampton, VA, USA) were mounted prior to filling the container with gel. All three sensors were equidistant to the entrance of the bolt tip in the gel sample (19 mm); transducer 1 was in the line of probe motion, transducer 2 was 30° and transducer 3 was 60° from the line of probe motion (see Figure S1 in Supplementary Material). The sensor signals were offset adjusted, amplified, and anti-aliasing filtered (200 kHz) using a DEWTRON DAQP-Bridge-B signal conditioner. The signals were digitized by a National Instrument USB-6251 data acquisition unit connected to a DELL laptop. This unit was controlled through the software LabView version 2013; referenced single ended measurements. Sample frequency was 333 kHz. An analog voltage sensitive trigger was used (triggered by transducer 1). The LabView program provided both unfiltered and filtered data; a low pass fourth order 10 kHz Butterworth filter was used.

#### Test procedures

Prior to the experiments with gel samples, the gel container ends were uncovered and any remaining gel in the bolt entrance holes was carefully removed. The container was firmly fixed to the test rig and positioned so that the projectile tip was in contact with the gel sample. The duration, the container was in laboratory room temperature prior to testing was <2 min. For the cavity study, tests were carried out at 50 and 100 bar rifle pressure using different bolt tip shapes (Table 2). For the pressure measurement study, tests were carried out at 100 bar air rifle pressure and with the flat shaped probe.

**Table 2:** **Gel test matrix and test results**.

No.	Probe shape			Pressure (bar)	Penetration (mm)	Probe velocity (m/s)	Maximum cavity diameter (mm)	Maximum cavity length (mm)
	**Sphere**	**Pencil**	**Flat**					
A1	*			50	5.2	108	7.3	7.5
A2	*			50	5.5	128	8.7	8.2
A3	*			50	5.5	124	8.4	7.6
B		*		50	5.7	100	2.2	5.5
C			*	50	5.3	121	9.7	8.1
D			*	100	5.1	143	12.4	9.5

## Results

### Lead bullet velocity and probe velocity

The bullet velocity for a 30, 50, and 100 bar rifle pressure was measured to 195 ± 0.9 meter per second (m/s) (*n* = 8), 242 ± 1.3 m/s (*n* = 12), and 314 ± 0.4 m/s (*n* = 8), respectively.

The maximum probe velocity was reached after approximately 1.5 mm probe travel into the brain tissue. The maximum of the spherical carbon fiber probe into the brain tissues was on average 87 ± 3 m/s (*n* = 7) when the rifle pressure was 50 bar. For identical conditions, the maximum velocity of the spherical aluminium probe was on average 110 ± 4 m/s (*n* = 3). When the air rifle pressure was 100 bar the maximum velocity of the flat aluminium probe was on average 146 m/s (*n* = 2).

### Pressure in the brain cavity during simulated penetration TBI

Maximum and minimum pressures, durations recorded in the animal experiments and probe penetrations are presented in Table S1 in Supplementary Material.

#### Distribution with flat probe tip

**Figure 3:**
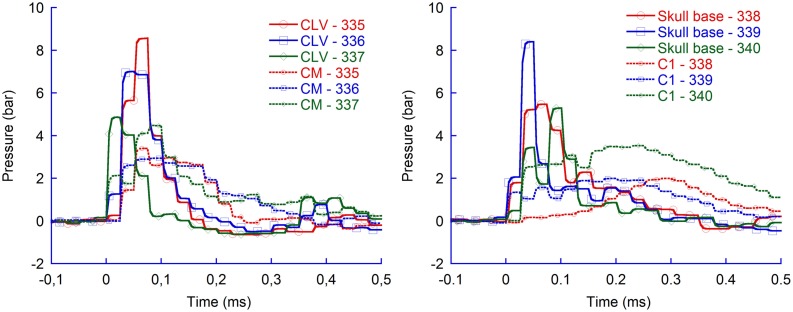
**Pressure recordings in four regions of rat brains during pTBI; contralateral ventricle (CLV), Cisterna Magna (CM), skull base, and upper cervical spinal canal (C1)**. Flat probe and 50 bar air rifle pressure.

During the probe penetration, the intracranial pressure increased by 4–9 bar (Figure 3). The average maximum pressure recorded in the CLV (6.8 bar) was significantly higher (*p* = 0.04) than the average recorded in CM (3.6 bar). However, the pressure was higher than 0.15 bar on average for only 0.2 millisecond (ms) in the CLV while 0.3 ms in the CM. Similarly, the average maximum pressure recorded in the skull base (6.4 bar) was statistically significantly higher (*p* = 0.02) than recorded in C1 (2.5 bar). Again the period with overpressure was shorter in the skull base region (0.2 ms) than in the C1 region (0.6 ms). Following the period of overpressure, the intracranial pressure dropped in all measured regions below ambient pressure; in some animals, the FOPT recorded as low absolute intracranial pressure as 0.4 bar relative ambient pressure in the CLV and in the skull base region. These drops lasted for 0.1 ms. The pressure drop in the C1 and CM regions, when flat probes were used, were negligible; the absolute pressure recorded was above 0.9 bar.

In addition, the flat probe produced pressures in the CLV of the same, or slightly greater magnitude, than those in the vicinity of the skull base. The results also demonstrate that the pressure changes were slighter in the CM and changed even less in the upper most spinal canal.

**Figure 4:**
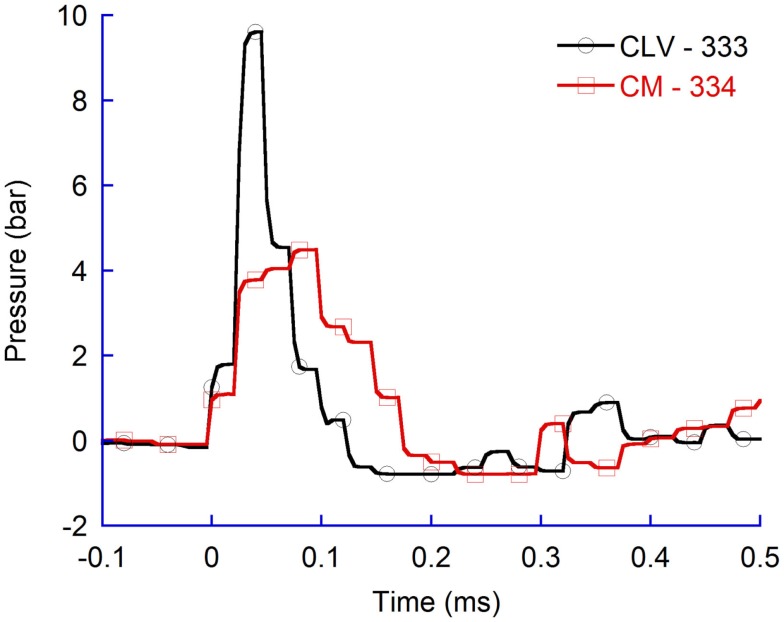
**Pressure recordings in contralateral ventricle (CLV) and Cisterna Magna (CM)**. Flat probes and 100 bar pressure.

Similarly, for higher air rifle pressures, resulting in faster probe velocity into the brain tissues, the maximum CLV pressure was higher than in the CM (Figure 4). The results verify that also at faster probe velocities the pressure propagates more efficiently to tissue volumes located in the direction of probe travel as compared to tissue volumes located more perpendicular to probe travel.

#### Distribution with spherical probe tip

**Figure 5:**
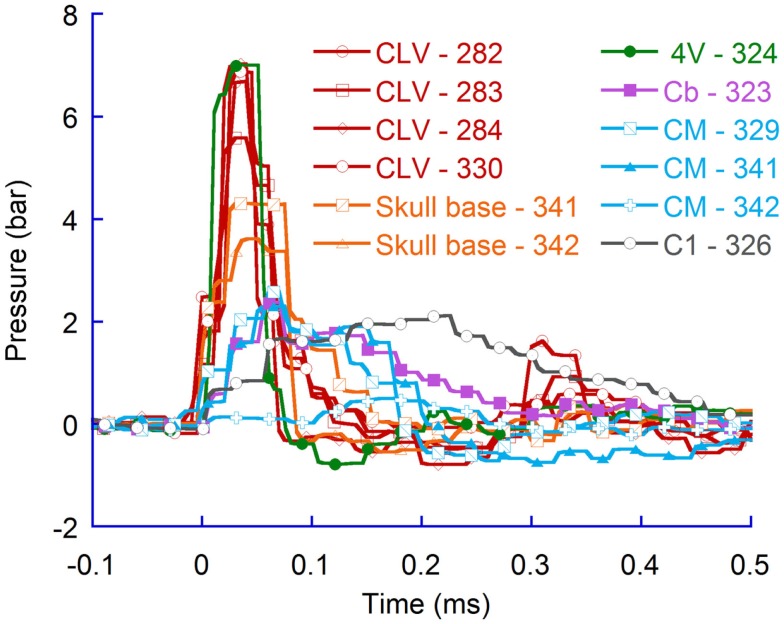
**Pressure recordings in the contralateral ventricle (CLV), the skull base, the fourth ventricle (4V), the middle of the cerebellum (Cb), the Cisterna Magna (CM), and the upper spinal canal (C1)**. Spherical probes at 50 bar rifle pressure.

Additional tests were carried out at 50 bar rifle pressure to assess if the pressure distribution pattern observed when flat probes were used would also be present for spherical probes (Figure 5). The results indicate that for spherical probes the pressure changes were also greater in the vicinity of the probe and lesser distal to the probe. Furthermore, the results confirm that the pressure propagated significantly more (*p* = 0.004) efficiently to tissue volumes located in the direction of probe travel (average maximum pressure change was 6.5 bar in the CLV) as compared to tissue volumes located more perpendicular to probe travel (average maximum pressure change was 1.8 bar in the CM). The results also confirm findings in experiments with flat probes; the periods when the pressure was above ambient pressure in the vicinity of the probe (on average 0.12 ms) were shorter than those distal to the probe (0.41 ms).

#### Effects of probe velocity

**Figure 6:**
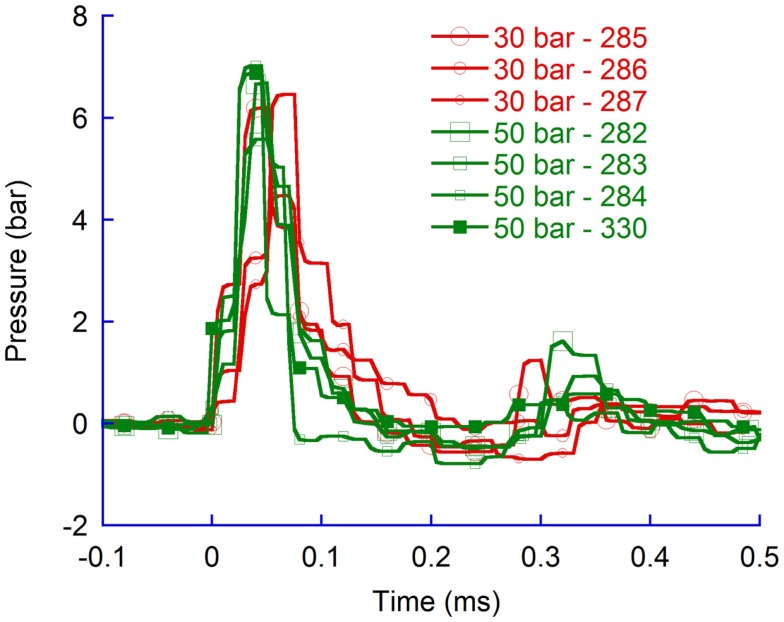
**Pressure recordings in the contralateral ventricle (CLV)**. Spherical probes at 30 and 50 bar air rifle pressure.

A 30 bar rifle pressure provided lower probe velocities and lower CLV pressure changes (5.7 bar) as compared to 50 bar rifle pressure changes (6.5 bar, spherical shaped aluminum probes). Although an apparent difference in pressure change was observed (Figure 6) the difference in average pressure change was not significant.

**Figure 7:**
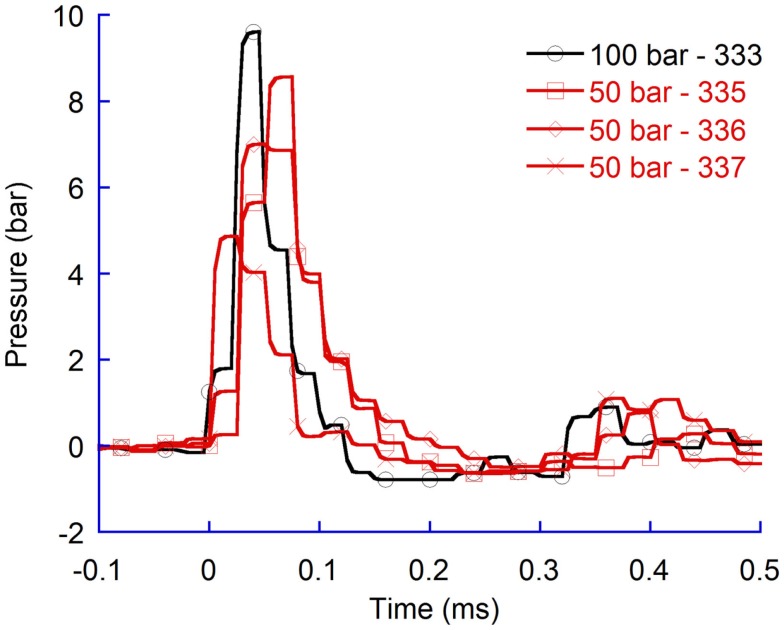
**Pressure recordings in the contralateral ventricle (CLV)**. Flat probes at 50 bar and 100 bar air rifle pressure.

A comparison of pressure time histories from tests with flat probes at 50 bar and 100 bar confirmed that a faster probe velocity, which was generated by increasing the air rifle pressure, generated greater pressure changes (Figure 7).

**Figure 8:**
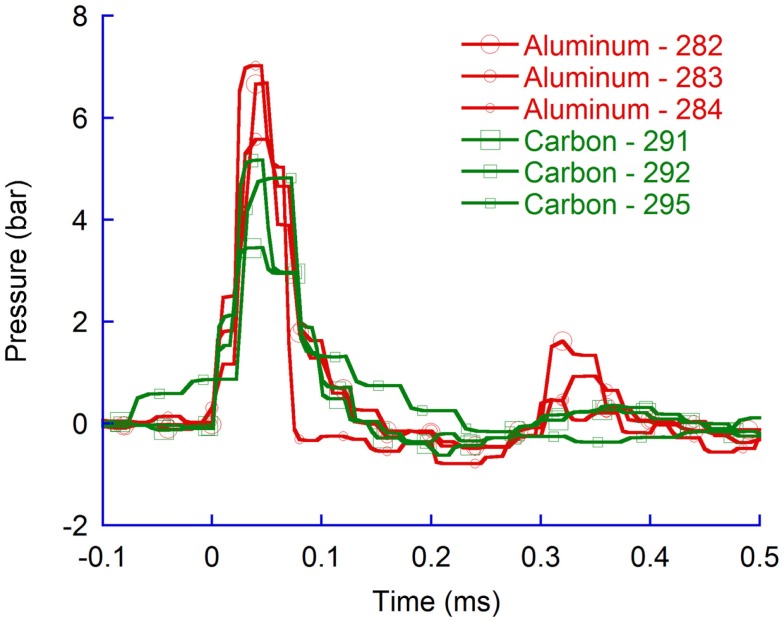
**Pressure recordings in the contralateral ventricle (CLV)**. Spherical probes in aluminum and in carbon fiber.

The carbon fiber probes were heavier than those constructed in aluminum and consequently the probe velocity was lower than for those made in aluminum. For identical probe-tip shapes, the average maximum pressure in the CLV were significantly higher (*p* = 0.02) for aluminum probes (6.5 bar) compared to carbon probes (4.5 bar) (Figure 8).

#### Effects of probe shape

**Figure 9:**
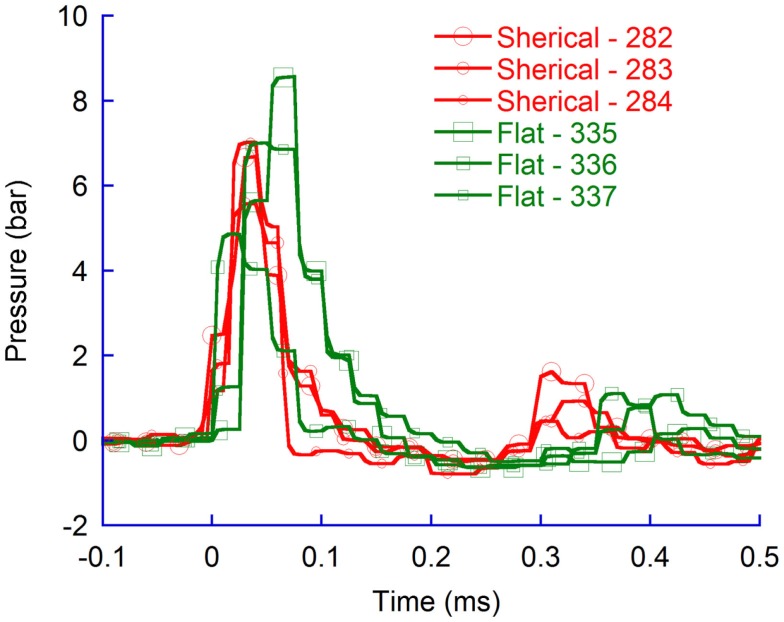
**Pressure recordings in the contralateral ventricle (CLV); spherical and flat probes**.

The spherical probe provided slightly lower maximum CLV pressure changes (6.5 bar) compared to the flat probe (6.8 bar) (Figure 9). The differences were not found to be significant.

**Figure 10:**
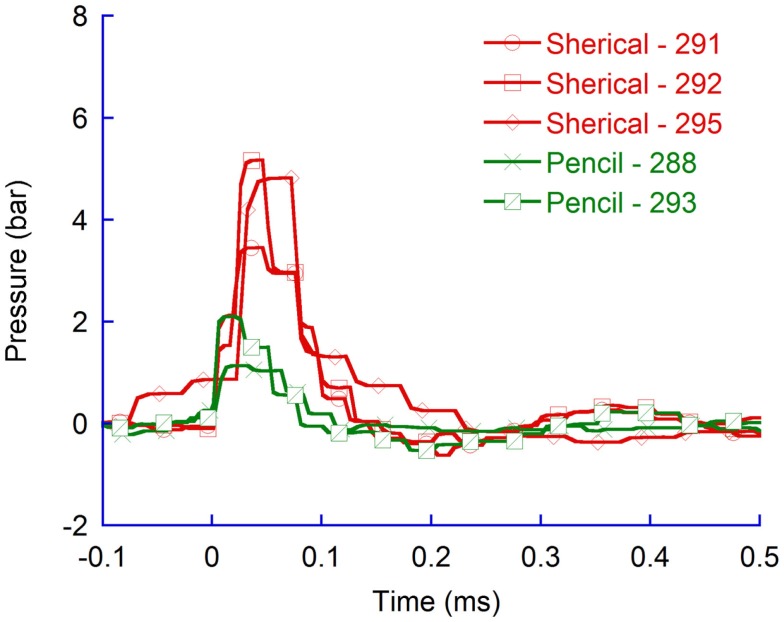
**Pressure recordings in the contralateral ventricle (CLV)**. Spherical and pencil shaped probes produced in carbon fiber.

Experiments carried out with pencil shaped probes produced in carbon provided significantly lower (*p* = 0.02) average maximum CLV pressures than spherical probes produced in carbon (Figure 10).

#### Comparing measurements with resistive transducer and FOPT

**Figure 11:**
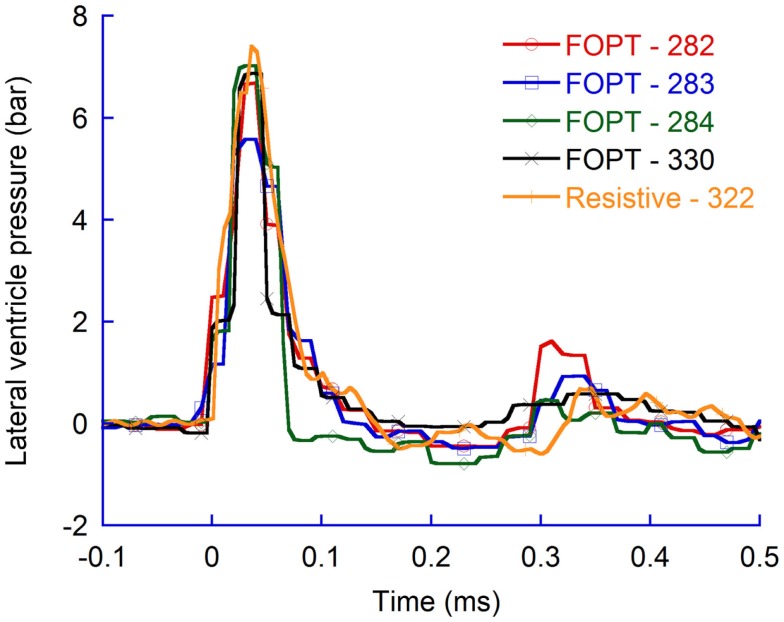
**Pressure recordings in the contralateral ventricle (CLV)**. FOPTs were used in 282, 283, 284, and 330 and resistive in 322.

The dimensions of the FOPT are such that it can be administrated into several brain regions with minimal effect on brain structure and pressure distribution. A larger transducer, such as a resistive transducer, only lends itself to pressure measurements in some brain regions. Nevertheless, experience of using the particular FOPT used in this study for brain pressure measurements in the brain cavity is limited and for this reason pressure measurements with a traditional resistive transducer connected to a regular measurement system was adopted to evaluate the FOPT measurement system. The results obtained with the resistive transducer and the measurement system applied indicate that the FOPT transducers and control unit used throughout this study provided useful data; the two systems measured similar pressure change during penetration trauma (Figure 11).

### Gel sample studies

#### Cavity formation in gel samples during simulated penetration TBI

**Figure 12:**
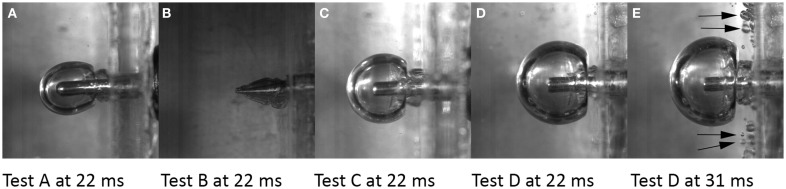
**Single photos of the experiments with the gel samples captured at 0.22 ms after probe motion start (A–D) and at maximum cavity (A–C,E)**. Arrows indicate cavities scattered in the gel outside the main cavity.

For a 50 bar air rifle pressure, the sphere shaped probe reached its maximum velocity of 120 ± 11 m/s after 1.5 mm probe travel (Table 2). Probe velocity was slightly less for the pencil shaped probe and similar for the flat probe. Images captured when the cavity was at its largest size in the gel experiments revealed that the flat probe produced larger cavities than the spherical probe while the pencil shaped probe produced very small cavities (Figures 12A–C). The cavity collapsed in all experiments; none or very little cavity remained. The gel samples appeared to be unaffected by the trauma; no structural changes or opacities could be observed following the trauma.

The rifle air pressure had an effect on the resulting probe velocity; for 50 and 100 bar rifle pressure the resulting flat probe velocities were 121 and 143 m/s, respectively. The resulting maximum height of the cavities for these test conditions were 9.7 and 12.4 mm (Figures 12C,E). Maximum cavity was formed 0.09 ms later when a 100 bar air rifle pressure was used as compared to a 50 bar (Figures 12C–E).

In test with flat probes, small temporary cavities appeared outside the main cavity (Figures 12D,E). These were most frequent when the air rifle pressure was 100 bar. For air rifle pressures of 50 bar, cavities appeared in the gel adjacent to the plastic wall. For air rifle pressures of 100 bar, cavities were also found elsewhere.

#### Pressure distribution in gel during simulated penetration TBI

Several tests were carried out with gel samples prepared for pressure measurements. Reproducibility was considered high; hence for simplicity reasons only results from one test are presented. A rifle pressure of 100 bar provided a probe velocity of 140 m/s and moved the flat probe 6.5 mm into the gel. A maximum pressure of 2.4 bar was recorded by the transducer 1, which was located in front of the probe and at an average distance of 16 mm from the probe front. Maximum pressure recorded in in the periphery were lower; transducers 2 and transducer 3 recorded 2.1 bar and 1.5 bar, respectively.

## Discussion

This study aimed at measuring the pressures arising when rat brains are exposed to high velocity penetration trauma by very thin pressure measurement probes. We found that the maximum intracranial pressure was high, in relation to other trauma models commonly used, and the pressure appear to be related to probe penetration velocity and probe shape. In addition, we found that the pressure propagates at a high rate to distal brain regions but the amplitude decays rapidly.

### Pressure distribution

During the simulated penetration trauma, a probe was made to penetrate the brain tissue at a high velocity for approximately 6 mm. The resulting pressure changes were recorded in several brain regions in the rat brains during simulated penetration trauma. The results indicate that the pressure increases were greater in the proximity to the probe as compared to regions distal to the probe. The data also indicate that the pressure changes were similar in volumes in front of the probe and in volumes adjacent to the probe; average maximum pressure change was 6.4 bar in the skull base and 6.8 in the CLV (flat tip, 50 bar air rifle pressure, *n* = 3). However, the skull base pressure was recorded at a distance of 5–11 mm from the probe tip while the CLV pressure was recorded at a distance of 4–6 mm. Had the pressure sensor in the skull base region been located closer to the probe tip it is expected that a higher pressure would have been recorded.

In addition, the pressure changes were less in the CM and even lesser in the most cranial region of the spinal canal. The amount of energy that travels to the posterior regions of the skull cavity appears to decay through dissipation. It is possible that the pressure energy is mostly transferred to the tissue in front of the probe (in the direction of probe travel); this finding could explain the smaller pressure changes recorded in the rear brain regions.

Further, the period when the pressure was above ambient pressure vary; in the proximity of the penetrating probe the periods were short, about 0.12 ms, while more distally from the probe the periods above ambient pressure were much longer, on average 0.58 ms long. The pressure appears to wear off as the pressure propagates in the brain tissue, and this is compensated by an increased duration.

Any liquid flow or any wave traveling in the brain during trauma would apply additional pressure on any surfaces perpendicular to the flow direction. The surfaces sensitive to pressure changes in the FOPT sensor and in the resistive sensor were almost parallel to any hypothetical flow inside the brain when these sensors were positioned in the CLV, CM, and near the skull base region. Hence, these sensors measured the pressure that is of primary importance to determining net loads on the tissue. However, when the FOPT was positioned in the center of the cerebellum (Cb), forth ventricle (4V), and in the spinal canal at the level of the first cervical vertebra (C1) the sensor surface sensitive to pressure was perpendicular to any hypothetical flow inside the brain. Due to this sensor direction, the dynamic pressure was recorded in these positions. The contribution of any liquid flow or wave traveling in the brain is, however, considered small.

### Comparing pressure observed in penetration trauma to other type of brain trauma

The maximum CLV pressure recorded during penetration trauma was higher than that recorded in rats during fluid percussion injury (FPI), severe controlled cortical impact (CCI), and severe weight drop injury (WDI) (11). In FP, the maximum CLV pressure was 1.65 bar, which is 24% of the maximum CLV pressure recorded in experiments with the spherical probes at 50 bar rifle pressure. The maximum pressure recorded in the CLV in the WDI and the CCI experiments was even lower; 6 and 10% of the maximum pressure measured in the penetration trauma. These differences in CLV pressures were expected as the brain in the study by Clausen and Hillered (11) was deformed at a further distance from the CLV in the CCI and WDI experiments as compared to the penetration trauma. In addition, the impactor in the CCI traveled at a velocity of 4 m/s while only 2.4 m/s in the WDI. Further, the motion into the brain was only 2.5 mm in the case of the CCI and WDI while on average 5.5 mm for the penetration test rig used in this study.

Another difference between the penetration trauma and some of the other TBI models is the duration of any dominant but temporary pressure increase. In the penetration trauma, a pressure increase was observed for about 0.08 ms while in FP this was reported to have been 14 ms. The difference in duration may explain why there was a high degree of brain stem involvement in the FPI experiments not seen in the pTBI.

### Modeling penetrating traumatic brain injuries

It is important to realize that the pTBI model is not an exact representation of penetrating TBIs in humans. One fundamental difference is that the test rig controls the depth of the lesion while for human penetrating TBI the projectile may enter and exits the skull cavity. According to a survey of veterans surviving penetrating brain injury sustained in the Vietnam War, 261 of 1,010 suffered from a “through-and-through” penetrating brain injury (12). Nonetheless, the limited size of the rat brain would make an unrestricted penetration very difficult. Projectiles that display entrance velocities within the range applied in the present study would penetrate through the head and create large exit wounds. Such practice would be considered unethical and a non-acceptable model for detailed studies of tissue reactions. Therefore, we decided to limit the penetration depth by using a mechanical stop for the projectile, which provides the option of evaluating severe lesion at a local, but not global, level. Nevertheless, the probe velocity profile in the pTBI model is, for 75% of its travel, fairly representative of a free-flying projectile traveling through a brain.

The pTBI could be considered a proper representation of pTBIs leaving shrapnel in the brain cavity. According to a 15-year follow-up study of 520 veterans surviving penetrating brain injury sustained in the Vietnam War, 66% retained metal fragments in the skull cavity (13). However, in our model, the probe travels to a pre-defined distance at a relatively constant velocity, approximately 75% of its traveling distance, into the brain until it rapidly comes to a full stop. As such the model does not fully reproduce a trauma in which a free-flying projectile lodges in the brain tissue. The projectile velocity in such a trauma would be expected to be reduced along its entire motion in the brain. Hence, the temporary cavity witnessed in the gel sample does not fully mimic the temporary cavity that would be seen if a free-flying projectile would lodge on its own. For such a free-flying projectile, the cavity would be likely to appear earlier in the wound tract and be less dramatic in front of the penetrating probe as was demonstrated in Figure 3. Further, any pressure change that arises in the brain cavity may be less intense than recorded in this study.

For a free-flying object to come to a full stop in a rat brain, without producing fatal brain injuries, the projectile must either be very light or its velocity upon brain contact rather low. The latter solution would provide projectile velocities less representative of shrapnel or bullet velocities. The former solution would either produce a rather narrow wound tract or, if the projectile is produced in low density material, may deviate from the preferred penetration path. Reproducibility between animal trials is expected to be lower than if the probe is guided.

The pTBI can be used to study severe injuries to important brain structures located in one of the brain hemispheres while the risk of fatality remains low. This model can also be used to analyze local effects of changes in velocity and form of the projectile. As a side effect, we can also evaluate how transient local pressure gradients are propagating to different parts of the brain. Such data are important for future work with FE-models. However, any direct comparisons to the outcome of pTBI in humans should be avoided until further analyses have been undertaken.

### Scaling the results to humans

Objects entering the human brain at high velocity, such as bullets and shrapnel, range considerably in size. In this study, a probe with a diameter of 2 mm was used. This probe size is smaller than common shrapnel fragments and bullets but is in relation to the size rat brains rather large. The probe made up about 17% of the brain width and the probe traveled some 60% of the brain height. Commonly, right circular cylinder (RCC) projectiles of sizes ranging from 2 to 64 grains are used in personal armor fragment tests. The diameter of the RCC 64 grains projectile is approximately 9% of the human brain width. Scaling laws facilitating scaling of the obtained results to be valid for humans are lacking. Hence, in the near future FE-models of the test set up will be used to scale the obtained results to humans.

### Correlation between pressure changes and pathology

In a previous study (9), we described progressive tissue destruction, including cavity formation, white matter degeneration, hemorrhage, edema, and gliosis in the tissue surrounding the penetrating probe. We also used a number of behavioral models to examine the neurological outcome, with the most noteworthy finding being impairment of reference memory function. This memory category represents knowledge of aspects of certain tasks that remain constant between trials. Impairment has been associated with thalamic injury (14). Thus, the data in the present study may facilitate the understanding of how physics affects pathology following this type of focal injury. Histological examinations showed that the trauma produced injuries remotely to the volume directly affected by the penetrating probe (Silver stained end bulbs in the internal capsule of the brain). However, these injuries were only found within the brain hemisphere where the probe entered and not in the contralateral hemisphere in which pressure was recorded in this study. Hence, combining the results in this study with those of Plantman et al. suggests pressure changes recorded in this study were non-injurious. Although we acknowledge that additional studies are needed to confirm this suggestion, we should also take into account other injury detection techniques may reveal injuries in the contralateral brain hemisphere or in the cerebellum and brainstem. Further, a higher probe velocity, more representative of those common in gun related injuries, may induce primary brain tissue injuries in brain regions distal to the wound tract.

### Cavity formation in gel samples and its relation to pressure changes and cell injuries

Temporary cavities of different sizes were observed in the experiments with gel samples in this study. Whether these cavities would also be formed in experiments with animals is currently unknown. However, it has been reported that when a bullet at high velocity enter live soft tissue a temporary cavity is formed (15). Collapse of the cavities is also expected to take place in live tissue; it has been reported that when a cavity in live tissue has reached its maximum dimension it collapses due to tissue elasticity (16, 17). Hence, the results obtained in the gel experiments and the findings in the above references indicate that cavitation is likely to occur when the pTBI test rig is used with animals. It is also likely that such a cavity collapses and may give rise to injuries similar to those we have observed (9, 18) in the tissues surrounding the probe although not having been in contact with the probe during trauma. This suggestion is supported by Risling et al. (19) who found that tissue degeneration and inflammation are more severe following high velocity pTBI (85 m/s) than after low velocity pTBI (1 m/s). This suggestion is also supported by Sondén et al. (5); based on their experiments that suggest that cell death in soft tissue not having been in direct contact with the penetrating bullet could be caused by the pressure wave that propagates through the tissue. However, a relationship between the temporary cavity size and cell death in the surrounding brain tissue has not been established (20).

In this study, any pressure wave propagation in the gel sample could not be observed. Such observations require a particular light setting, which was not adopted in the experiments with gel samples. However, a pressure increase by approximately 1.4 bar followed by virtually vacuum was recorded at an average distance of 13 mm from the probe. Hence it is likely that a pressure wave was generated as a consequence of the formation of a temporary cavity.

The shape of the cavities observed in the gel experiments was almost spherical when the flat and the spherical probes were used. The cavity center was somewhat behind the probe front. For the flat probe at 50 bar rifle pressure, the probe front was 1.4 mm ahead of the cavity center. For the flat probe at 100 bar rifle pressure, the probe front was 3.7 mm ahead of the cavity center. The spherical shape may indicate that gel pressure changes would be identical at any given distance from the bolt tip at the velocities in this study. However, the cavity wall travels momentarily at a greater velocity in the direction of probe travel compared to cavity wall velocity perpendicular to probe travel. As a result, it can be speculated that any pressure increase at some distance from the cavity varies with the position relative to the probe. It can also be expected that pressure fluctuations in volumes located in the path of the probe will be greater than those located more peripherally.

Shrapnel and bullet velocities vary but can be much higher than those used in these experiments. The test conditions in the cavity formation study using gel samples, i.e., probe velocities, probe dimensions, and probe distance traveled into the gel, were chosen as these test conditions have been used extensively in past experiments with animals. In addition, when these test conditions were used in animal experiments, injuries were produced in volumes suitable for brain injury studies. Experiments carried out with higher probe velocities produce extensive brain damage that commonly terminates a life within seconds following trauma; the results would be of very limited value.

Cavity formation was found to be related to probe impact shape as well as the maximum pressure recorded in the CLV in experiments with animals. The pencil probe produced the smallest cavity and also the least maximum pressure in the CLV while the flat probe produced largest cavity and greatest maximum pressure in the CLV. Too few measurement points were available to draw definitive conclusions, but the results indicate cavity size and/or cavity wall velocity is correlated with the amplitude of the pressure increase and drop in the brain.

### Kinetic energy

Commonly, the kinetic energy transferred to the tissue is provided in studies of projectile behavior when traveling through tissues or gel samples. For the experiments carried out in this study, it is not easy to calculate the kinetic energy; energy is transferred to a stopping device and to the gel or the animal brain.

### Future studies

Finite element models are complex mathematical formulas that mimic reality. Future studies include the use of FE-models of the animal head and trauma model to improve our understanding of the relationship between the characteristics of the penetrating probe and pressure. In the longer term, when additional pathology data are made available, these FE-models can be used to correlate internal parameter values, e.g., delivered pressure impulses, to tissue pathology. Such a model can also be used to compare the trauma model to clinical trauma.

An improved understanding of the relationship between internal parameter values and injury will also be extremely valuable in the development of FE-models of the human head would be useful tools in the design of protection against penetrating brain trauma

## Conclusion

In this study, probes were used to penetrate animal brains while recording pressure changes in different regions of the brain and spinal canal. It was concluded probe impact generated short lasting pressure pulses in the brain tissue. Overpressure was as high as 8 bar in this animal model modified to simulate penetration trauma. Following overpressure the intracranial pressure dropped to as low as 0.4 bar relative to ambient pressure recorded in the CLV.

Pressure pulses were of similar magnitude in the CLV and in the vicinity of the skull base. The pressure pulses in the periphery of the penetration, in the CM, were significantly less powerful than the pulses recorded in the vicinity and in front of the penetrating probe.

In addition, this study characterized the cavity produced during probe penetration into ballistic gel samples. High-speed videos revealed temporary gel cavities; the cavity size and wall velocity may correlate with the pressure changes recorded in the CLV in the animal brains.

## Conflict of Interest Statement

The authors declare that the research was conducted in the absence of any commercial or financial relationships that could be construed as a potential conflict of interest.

## Supplementary Material

The Supplementary Material for this article can be found online at http://www.frontiersin.org/Journal/10.3389/fneur.2015.00051/abstract

The rifle pressure, probe type, approximate probe velocity, and maximum pressure are presented in Table S1 in Supplementary Material. The containers used for casting and holding the gel samples during testing were constructed in plastic. The dimensions of the containers are presented in Figure S1 in Supplementary Material.
