# Supplementary material for: Characterization of Pressure Distribution in Penetrating Traumatic Brain Injuries
Source: Front Neurol. 2015 Mar 13;6:51. doi: 10.3389/fneur.2015.00051 (PMC4358068; doi:10.3389/fneur.2015.00051)
Supplement: Supplementary file 1 [file table_1.docx]

| No. | Pressure (bar) | Probe velocity^2^  (m/s) | Penetration  (mm) | Transducer position | Maximum pressure  (bar) | Duration of maximum pressure greater than 0.15 bar (ms) | | Minimum pressure  (bar) | | Duration of minimum pressure greater than -0.15 bar (ms) | |  |
| --- | --- | --- | --- | --- | --- | --- | --- | --- | --- | --- | --- | --- |
| Spherical shaped aluminum probe | | | | | | | | | | | | |
| 285 | 30 | na | 5.8 | CLV | 6.2 | 0.13 | -0.6 | | 0.13 | |  |  |
| 286 | 30 | na | 5.7 | CLV | 4.5 | 0.21 | -0.2 | | 0.09 | |  |  |
| 287 | 30 | na | 5.7 | CLV | 6.5 | 0.16 | -0.7 | | 0.15 | |  |  |
| 282 | 50 | 110 | 5.7 | CLV | 6.7 | 0.15 | -0.5 | | 0.13 | |  |  |
| 283 | 50 | 110 | 5.7 | CLV | 5.6 | 0.13 | -0.5 | | 0.15 | |  |  |
| 284 | 50 | 110 | 5.7 | CLV | 7.0 | 0.07 | -0.8 | | 0.22 | |  |  |
| 330 | 50 | 110 | 6.0 | CLV | 6.9 | 0.15 | -0.4 | | 0.15 | |  |  |
| 322 | 50 | 110 | 5.9 | CLV^1^ | 7.4 | 0.14 | -0.6 | | 0.17 | |  |  |
| 329 | 50 | 110 | 5.8 | CM | 2.6 | 0.18 | -0.7 | | 0.13 | |  |  |
| 341 | 50 | 110 | 6.2 | CM | 2.3 | 0.20 | -0.7 | | 0.29 | |  |  |
| 341 | 50 | 110 | 6.2 | Skull base | 4.3 | 0.16 | -0.3 | | 0.12 | |  |  |
| 342 | 50 | 110 | 6.4 | CM | 0.5 | 0.27 | -0.2 | | 0.02 | |  |  |
| 342 | 50 | 110 | 6.4 | Skull base | 3.6 | 0.08 | -0.5 | | 0.14 | |  |  |
| 323 | 50 | 110 | 5.9 | Cb | 2.4 | 0.49 | -0.2 | | 0.21 | |  |  |
| 324 | 50 | 110 | 5.9 | 4V | 7.0 | 0.74 | -0.8 | | 0.11 | |  |  |
| 326 | 50 | 110 | 5.9 | C1 | 2.1 | 0.57 | -0.3 | | 1.20 | |  |  |
| Flat shaped aluminum probe | | | | | | | | | | | | |
| 335 | 50 | 110 | 6.3 | CLV | 8.6 | 0.16 | | -0.6 | | 0.18 | |  |
| 335 | 50 | 110 | 6.3 | CM | 3.4 | 0.22 | | -0.4 | | 0.20 | |  |
| 336 | 50 | 110 | 6.4 | CLV | 7.0 | 0.20 | | -0.5 | | 0.13 | |  |
| 336 | 50 | 110 | 6.4 | CM | 2.9 | 0.34 | | -0.2 | | 0.05 | |  |
| 337 | 50 | 110 | 6.2 | CLV | 4.9 | 0.15 | | -0.6 | | 0.20 | |  |
| 337 | 50 | 110 | 6.2 | CM | 4.5 | 0.42 | | -0.2 | | 0.56 | |  |
| 338 | 50 | 110 | 6.2 | Skull base | 5.5 | 0.32 | | -0.4 | | 0.11 | |  |
| 338 | 50 | 110 | 6.2 | C1 | 2.0 | 0.62 | | -0.1 | | na | |  |
| 339 | 50 | 110 | 6.2 | Skull base | 8.4 | 0.16 | | -0.4 | | 0.17 | |  |
| 339 | 50 | 110 | 6.2 | C1 | 2.0 | 0.48 | | -0.1 | | na | |  |
| 340 | 50 | 110 | 6.3 | Skull base | 5.3 | 0.13 | | -0.3 | | 0.11 | |  |
| 340 | 50 | 110 | 6.3 | C1 | 3.6 | 0.64 | | -0.1 | | na | |  |
| 333 | 100 | 146 | 6.1 | CLV | 9.6 | 0.13 | | -0.8 | | 0.20 | |  |
| 334 | 100 | 146 | 6.1 | CM | 4.5 | 0.17 | | -0.8 | | 0.15 | |  |
| Spherical shaped carbon fibre probe | | | | | | | | | | | | |
| 291 | 50 | 87 | 5.0 | CLV | 3.5 | 0.13 | | -0.6 | | 0.12 | |  |
| 292 | 50 | 87 | 5.2 | CLV | 5.2 | 0.14 | | -0.4 | | 0.12 | |  |
| 295 | 50 | 87 | 5.1 | CLV | 4.8 | 0.22 | | -0.4 | | 0.16 | |  |
| Pencil shaped carbon fibre probe | | | | | | | | | | | | |
| 288 | 50 | 87 | 6.9 | CLV | 1.1 | 0.12 | | -0.2 | | 0.05 | |  |
| 293 | 50 | 87 | 5.8 | CLV | 2.1 | 0.09 | | -0.5 | | 0.19 | |  |
| 1 Resistive transducer | | | | | | | | | | | |  |
| 2 Estimated | | | | | | | | | | | |  |
| na Not available | | | | | | | | | | | |  |
