# Supplementary figures and images for: Characterization of Pressure Distribution in Penetrating Traumatic Brain Injuries
Source: Front Neurol. 2015 Mar 13;6:51. doi: 10.3389/fneur.2015.00051 (PMC4358068; doi:10.3389/fneur.2015.00051)

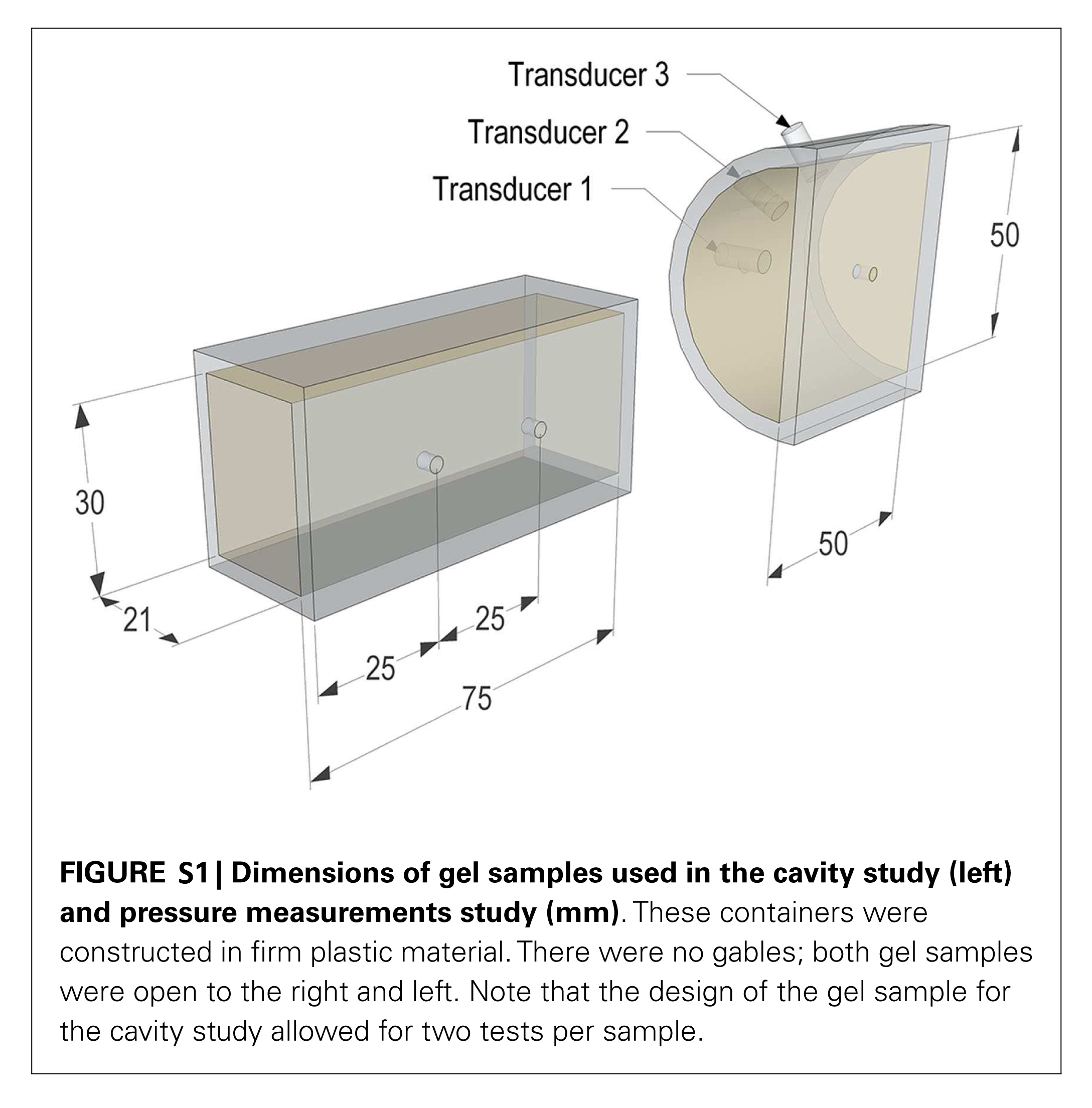

Supplement: Supplementary file 2 [file image_1.jpeg]
